# Teprotumumab for Thyroid Eye Disease: Mechanism, Clinical Efficacy, and Current Challenges

**DOI:** 10.3390/antib14030055

**Published:** 2025-06-30

**Authors:** Yuan Zong, Shuang Qiu, Mingming Yang, Jing Zhang, Yaru Zou, Yuxin Jing, Kyoko Ohno-Matsui, Koju Kamoi

**Affiliations:** 1 Department of Ophthalmology, Zhongshan Torch Development Zone People’s Hospital, Zhongshan 528436, China; zongyuan666@gmail.com (Y.Z.); 18925301120@163.com (S.Q.); 2 Department of Ophthalmology & Visual Science, Graduate School of Medical and Dental Sciences, Institute of Science Tokyo, Tokyo 113-8510, Japan; yangmm-12@outlook.com (M.Y.); zhangj.c@foxmail.com (J.Z.); alicezouyaru519@gmail.com (Y.Z.); k.ohno.oph@tmd.ac.jp (K.O.-M.); 3 International Ocular Surface Research Center, Institute of Ophthalmology, and Key Laboratory for Regenerative Medicine, Jinan University Medical School, Guangzhou 510632, China; mintchocolate_0417@163.com; 4 Department of Ophthalmology, The First Affiliated Hospital of Jinan University, Guangzhou 510630, China

**Keywords:** clinical efficacy, Graves’ disease, insulin-like growth factor-1 receptor, orbital fibroblasts, teprotumumab, thyroid eye disease

## Abstract

Thyroid eye disease (TED) is a complex autoimmune disorder characterized by orbital inflammation and tissue remodeling. Teprotumumab, a fully human monoclonal antibody targeting insulin-like growth factor-1 receptor (IGF-1R), represents a significant breakthrough in TED treatment. This review comprehensively analyzes the therapeutic role of teprotumumab in TED management. Mechanistically, teprotumumab inhibits the IGF-1R/TSHR signaling complex, thereby reducing orbital fibroblast differentiation and inflammatory responses. Phase II and III clinical trials have demonstrated its remarkable efficacy in reducing proptosis and improving clinical activity scores, with the benefits extending to both active and chronic TED cases. Real-world studies have validated these findings further and expanded its potential applications to various clinical scenarios, including dysthyroid optic neuropathy and steroid-resistant cases. However, several challenges remain. These include treatment-related adverse effects such as hyperglycemia and hearing impairment, with emerging evidence suggesting ethnic variations in susceptibility. The high cost of treatment poses significant accessibility barriers, while limited long-term follow-up data and potential disease recurrence necessitate further investigation. This review synthesizes the current evidence to inform clinical decision-making and highlights areas requiring additional research to optimize teprotumumab’s therapeutic application in TED management.

## 1. Introduction

Thyroid eye disease (TED), also known as thyroid-associated ophthalmopathy (TAO) or Graves’ orbitopathy (GO), represents an uncommon inflammatory condition affecting the orbital tissues (with an estimated overall prevalence of approximately 0.25%), characterized by lymphocytic infiltration, expansion of the orbital adipose tissue, and enlargement of the extraocular muscles [1,2,3]. This condition exhibits a strong association with Graves’ disease (GD), with approximately 40% of GD patients developing varying degrees of ocular involvement [2]. The clinical significance of TED lies in its potential to compromise visual function, induce functional impairments, and cause substantial disfigurement, accompanied by consequential psychosocial sequelae [2,4,5]. Despite extensive research efforts, the precise pathophysiology, preventive strategies, and optimal therapeutic approaches for TED remain incompletely elucidated [1]. The current therapeutic approaches to TED primarily encompass corticosteroid therapy, radiotherapy, surgical intervention, and immunosuppressive treatment [1]. However, these conventional treatment modalities generally exhibit suboptimal efficacy, significant adverse effects, and limited applicability. Notably, corticosteroid monotherapy fails to effectively inhibit disease progression, particularly in terms of proptosis development, often necessitating combination therapy with multiple treatment strategies [1,4,5].

This review addresses a critical question: How does teprotumumab, as the first FDA-approved targeted therapy for TED, impact the treatment landscape in terms of its mechanism, clinical efficacy, and implementation challenges? Teprotumumab (TEPEZZA^®^) is a fully human monoclonal antibody targeting insulin-like growth factor-1 receptor (IGF-1R). IGF-1R overactivation, induced by autoimmune responses in TED patients, is seen in the orbital fibroblasts (OFs) in both active and inactive TED cases [2,6,7]. This receptor forms functional complexes with thyroid-stimulating hormone receptor (TSHR) on the cell membranes of OFs, B cells, and T cells, thereby promoting the characteristic inflammatory processes and tissue remodeling observed in TED. The overexpression of IGF-1R is considered a pivotal pathophysiological mechanism underlying the development and progression of TED [4,7]. Currently, teprotumumab has demonstrated efficacy in a series of clinical trials for both acute and chronic TED treatment and has received U.S. Food and Drug Administration (FDA) approval for TED therapy in the United States [2,6,7,8]. While recent reviews have examined specific aspects of teprotumumab therapy [9,10,11], our review integrates the underlying pathophysiology of TED with teprotumumab’s pharmacological properties, synthesizes the evidence from both clinical trials and real-world applications, and critically examines the current challenges including safety concerns, economic considerations, and treatment durability. This comprehensive analysis aims to better inform clinical decision-making and future research directions in TED management.

## 2. Methods

This review aimed to comprehensively evaluate teprotumumab’s role in thyroid eye disease (TED) treatment. We conducted a literature search from inception to April 2025 using PubMed, Web of Science, and Google Scholar. The search terms included “teprotumumab”, “thyroid eye disease”, “Graves’ orbitopathy”, and “thyroid-associated ophthalmopathy”. Additional relevant articles were identified through reference lists of retrieved publications and FDA documentation related to teprotumumab’s approval.

We included English-language publications focusing on teprotumumab for TED treatment, encompassing clinical trials, observational studies, case series, and review articles. Studies addressing the pathophysiology of TED, the mechanism of action of teprotumumab, clinical outcomes, and safety profiles were considered relevant for inclusion. We excluded articles not directly related to teprotumumab or TED.

The data extraction focused on the study characteristics, patient populations, treatment protocols, clinical outcomes (particularly proptosis reductions, clinical activity score improvements, and changes in diplopia), adverse events, and economic considerations. Given the evolving nature of the field and the relatively recent approval of teprotumumab, we included both peer-reviewed publications and high-quality data from conference proceedings when necessary to provide the most current information.

This review synthesizes the available evidence in a narrative format, presenting the current understanding of teprotumumab’s efficacy, safety, and practical implementation challenges in TED management.

## 3. The Pathophysiology of Thyroid Eye Disease

**Figure 1:**
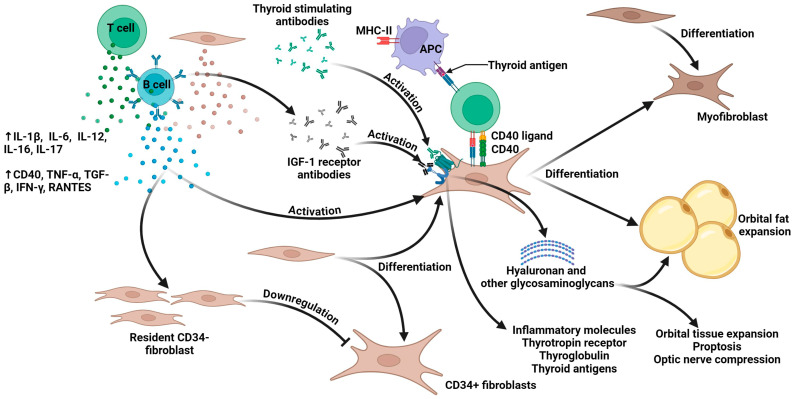
Pathophysiology of thyroid eye disease (TED).

GD is frequently associated with ocular disorders, among which TED is the most well-recognized as an autoimmune condition [7,12,13,14,15,16]. However, a small number of TED patients exhibit normal or reduced thyroid function [1]. The primary causative factor of GD stems from the activation of TSHR autoantibodies. However, the pathogenesis of TED involves more intricate mechanisms, with complex interactions among genetic predisposition, environmental factors, and immune system dysregulation [1,5]. The most common pathological anatomical feature in TED patients is enlargement of the extraocular muscles and retro-orbital fat/connective tissue compartments [17]. Figure 1 demonstrates the potential pathophysiological mechanisms underlying thyroid eye disease.

This figure illustrates the theoretical model of TED’s pathogenesis, highlighting the pivotal role of the orbital fibroblasts (OFs) as effector cells. The cascade initiates with immune activation involving the T and B cells, which release multiple pro-inflammatory cytokines (IL-1β, IL-6, IL-12, IL-16, IL-17, CD40, TNF-α, TGF-β, IFN-γ, and RANTES). The B cells generate TSHR antibodies and IGF-1R antibodies. The central pathway demonstrates antigen-presenting cells (APCs) presenting thyroid antigens via MHC-II molecules, activating the T cells through CD40-CD40 ligand interactions. These activated immune cells trigger OF activation through multiple mechanisms. The diagram depicts two distinct fibroblast populations: CD34^+^ fibroblasts (derived from fibrocytes) and resident CD34^−^ fibroblasts. CD34^−^ fibroblasts produce Slit2, which downregulates the gene expression in the CD34^+^ fibroblasts. CD34^+^ fibroblasts can differentiate into myofibroblasts or adipocytes. Activated OFs synthesize HA and other GAGs while expressing inflammatory molecules including TSHR, thyroglobulin, and thyroid antigens. Collectively, these processes contribute to orbital tissue expansion, proptosis, and optic nerve compression (adapted with permission from Scarabosio et al., 2024, Ref. [5], CC BY 4.0 License; see https://creativecommons.org/licenses/by/4.0/; accessed on 25 May 2025).

It is currently believed that the main effector cells responsible for the enlargement of the orbital soft tissues in TED are the OFs located in the interstitial spaces of the orbital muscle fibers, fat, and connective tissue. These are a unique group of CD34^+^ fibroblasts derived from the bone marrow, characterized by their unique phenotypic features, including high sensitivity to cytokines and distinctive tissue remodeling capabilities [1,5,18,19].

At the molecular level, two critical autoimmune targets have been identified: TSHR and IGF-1R. IGF-1R is a transmembrane tyrosine kinase receptor widely expressed in various cell types during fetal development and postnatally, where it performs essential physiological functions. When activated by IGF-1 and IGF-2, IGF-1R mediates cellular proliferation responses, serving as the primary pathway for mammalian somatic growth during fetal development and working synergistically with growth hormone after birth. Additionally, IGF-1R acts as a “cell survival factor”, protecting the neurons, hematopoietic cells, and fibroblasts from programmed cell death. In terms of signal transduction, IGF-1R operates through the PI3K-Akt/PKB pathway (leading to Bad phosphorylation, the induction of anti-apoptotic protein expression, the prevention of cytochrome c release, and inhibition of caspase activation) and the Ras-MAPK pathway (through adaptor proteins Shc and IRS-1/2, activating Ras and initiating a phosphorylation cascade that ultimately activates extracellular signal-regulated kinases 1/2). Furthermore, IGF-1R stimulates the differentiation of pre-adipocytes and myoblasts while playing crucial roles in pancreatic β-cell neogenesis and mammary gland development. In cochlear homeostasis, IGF-1 binds to IGF-1R to activate downstream signaling pathways (such as PI3K-AKT and MAPK) that regulate the survival, differentiation, and homeostasis of cochlear cells [20,21,22,23]. The complexity of these systemic effects raises significant concerns about potential systemic adverse effects when employing targeted therapies directed against IGF-1R.

These receptors demonstrate functional crosstalk through co-localization on the OFs, leading to synergistic cellular responses [24]. The formation of a functional complex between IGF-1R and TSH-R represents a critical trigger point in TED’s pathogenesis, directly activating OFs and leading to the abnormal secretion of pro-inflammatory cytokines and glycosaminoglycans. This abnormal secretion results in excessive deposition of glycosaminoglycans in the orbital tissues, while the pro-inflammatory cytokines trigger local inflammatory cascades and immune cell infiltration, collectively leading to orbital tissue edema and fibrotic process [1]. OFs can be divided into two subpopulations based on the expression of the surface marker Thy1 (also known as CD90) [25,26]. These subpopulations exhibit distinct differentiation patterns following activation: Thy1-expressing (Thy1^+^) OFs, primarily located in the muscle membranes of the extraocular muscles, differentiate into myofibroblasts and trigger muscle hypertrophy, while producing higher levels of prostaglandin-related molecules [17,27,28]. In contrast, Thy1-nonexpressing (Thy1^−^) OFs, widely distributed throughout the orbit, differentiate into mature adipocytes, leading to orbital fat tissue expansion, while producing more interleukin (IL)-8 [26,28]. In TED, the relative proportions of activated Thy1^+^ and Thy1^−^ OFs may determine the dominance of fibrosis versus adipogenesis in the lesions [25].

Immune cells including T cells, B lymphocytes, plasma cells, macrophages, and mast cells infiltrate the orbital tissues. These immune cells, in conjunction with OFs, secrete various inflammatory mediators, such as interferon-γ (IFN-γ), IL-1, IL-6, IL-8, and transforming growth factor-β (TGF-β), thereby triggering inflammatory responses. These mechanisms constitute significant pathogenic components in the development of TED. Consequently, targeting these immune pathways represents potential therapeutic strategies for TED [1].

For instance, rituximab, a chimeric murine–human monoclonal antibody, targets the CD20 antigen on B cells. It modulates B-cell antigen presentation and inhibits the production of TSHR-stimulating antibodies (TSAbs) and cytokines by the B cells [29]. IL-6 plays multiple roles in TED’s pathogenesis by upregulating the TSHR expression in the orbital tissues, promoting B-cell proliferation and differentiation into antibody-producing plasma cells, and facilitating the differentiation of OFs into myofibroblasts and mature adipocytes. Tocilizumab, a humanized anti-IL-6 receptor monoclonal antibody, competitively blocks IL-6 binding to its receptor, thereby inhibiting intracellular signaling cascades and suppressing the pro-inflammatory effects of IL-6 [17,30].

The continuous accumulation of these pathological changes ultimately leads to an imbalance in the volume and pressure of orbital contents, clinically manifesting as progressive exophthalmos, restricted extraocular muscle movement, and venous return obstruction, which, in severe cases, can cause irreversible vision loss due to optic nerve compression [1,31].

## 4. The Traditional Clinical Management of TED

The traditional clinical management of TED before teprotumumab’s approval is summarized in Table 1. Briefly, TED can be categorized into an active phase characterized predominantly by inflammatory symptoms, followed by inactive and chronic stages. Under the European Group on Graves’ Orbitopathy (EUGOGO)’s clinical recommendations, intravenous methylprednisolone pulse (IVMP) therapy, either as monotherapy or in combination with mycophenolate sodium, constitutes the primary therapeutic approach to active Graves’ orbitopathy [32]. In cases of treatment failure, alternative therapeutic options include more targeted approaches such as anti-IL-6 (tocilizumab) or anti-CD20 (rituximab) agents. During the inactive phase, surgical intervention remains the mainstay of treatment, potentially encompassing orbital decompression, strabismus surgery, and eyelid procedures, depending on the patient’s specific symptomatology [32].

## 5. The Pharmacology of Teprotumumab

Teprotumumab, an antagonist of IGF-IR, demonstrates high affinity and specificity by binding to the cysteine-rich α-subunit of the extracellular domain, thereby blocking the activation and signal transduction of the TSHR/IGF-IR complex. Currently validated mechanisms include the inhibition of the TSHR/IGF-IR complex signaling pathway, which suppresses the hyaluronic acid production by the OFs, consequently reducing the volume of the extraocular muscle and orbital fat; the inhibition of CD34^+^ T-cell expression of costimulatory molecules such as CD80, CD86, major histocompatibility complex II (MHC II), and programmed death-ligand 1 (PD-L1), preventing T-lymphocyte activation; and reductions in the levels of IGF-IR and TSH-dependent phosphorylated AKT, suppressing TSH-induced expression of IL-6 and IL-8 in the OFs while indirectly inhibiting the release of pro-inflammatory cytokines, including IFN-γ and TNF-α, from the T and B lymphocytes [8,33,34,35]. Therefore, teprotumumab can alleviate the expansion, fibrosis, and inflammatory response in the orbital tissues in patients with TED, ultimately relieving the symptoms of TED [8,19,25].

Basic studies have provided molecular evidence supporting this mechanism of action and elucidating the cellular effects of teprotumumab in TED, encompassing anti-inflammatory, anti-fibrotic, and immunomodulatory properties. Chen et al. demonstrated through in vitro experiments that teprotumumab effectively reduces the cell surface expression of both IGF-1R and TSHR in fibroblasts derived from GD patients [34]. Furthermore, the treatment significantly attenuated the TSH-induced mRNA expression and protein production of IL-6 and IL-8 while also inhibiting TSH-dependent Akt phosphorylation [34]. These findings provide molecular evidence supporting the therapeutic potential of teprotumumab in TED by targeting the crosstalk between the IGF-1R and TSHR signaling pathways. Krieger et al. demonstrated through in vitro experiments that teprotumumab dose-dependently inhibits TED-Ig-induced hyaluronan secretion in OFs from patients with TED by blocking the interaction between TSHR and IGF-1R, without directly binding to TSHR [36]. Fernando et al. reported that insulin-like growth factor-I receptor inhibitors, including teprotumumab, significantly reduce the expression of major histocompatibility complex II (MHC II) and B7 proteins in the fibroblasts both in vitro and in vivo [35]. This may potentially reduce the T-cell activation and cytokine production in TED, thereby restoring immune tolerance.

## 6. Clinical Translation of Teprotumumab: From Trials to Practice

### 6.1. Evidence from Pivotal Clinical Trials and FDA Approval

The standard treatment regimen with teprotumumab involves intravenous administration (with an initial dose of 10 mg/kg, followed by subsequent doses of 20 mg/kg), administered every three weeks for a total of eight infusions [4,8,37]. In a pharmacokinetic evaluation, Xin et al. employed a population pharmacokinetic approach integrating data from phase I, II, and III clinical trials to characterize the linear pharmacokinetic profile of teprotumumab [38]. The analysis revealed its favorable pharmacokinetic properties, including low systemic clearance (0.334 L/day), prolonged elimination half-life (19.9 days), and limited volume of distribution. This study demonstrated that the approved dosing regimen for TED maintains an IGF-1R saturation above 90% throughout the dosing interval. No significant correlations were observed between drug exposure and either efficacy or safety endpoints. These findings collectively suggest that the current dosing regimen provides significant therapeutic efficacy with favorable tolerability in the treatment of TED.

Two multicenter, double-blind, randomized, placebo-controlled trials in the US and Europe—a phase II study published in 2017 [8] and a phase III “OPTIC” trial published in 2020 [7]—demonstrated that teprotumumab exhibited significant efficacy in reducing proptosis and clinical activity scores (CASs) while improving the quality of life in patients with active TED. The treatment demonstrated a favorable safety profile with manageable adverse events [7,8]. The phase II trial [8] enrolled 88 patients with active, moderate to severe TED. The participants were randomized to receive intravenous infusions of teprotumumab or a placebo for 8 infusions. At week 24, the primary endpoint (defined as both a ≥2-point reduction in the CAS and a ≥2 mm reduction in proptosis) was achieved by 69% (29/42) of the teprotumumab-treated patients, compared with only 20% (9/45) in the placebo group (*p* < 0.001). The teprotumumab group demonstrated significantly greater mean reductions in proptosis (*p* < 0.001), with 40% of these patients achieving ≥4 mm reductions versus 0% in the placebo group. Teprotumumab treatment also resulted in significantly greater mean CAS reductions at all of the time points assessed (*p* < 0.001). The Graves’ Orbitopathy-Specific Quality of Life (GO-QoL) visual functioning scores improved significantly more in the teprotumumab group, exceeding those for the placebo group by 12.8 to 15.6 points. The composite GO-QoL scores showed significant improvements at weeks 6, 12, and 24 (*p* = 0.003, 0.007, and 0.012, respectively). Regarding safety, the adverse events that occurred in >5% of teprotumumab-treated patients, and more frequently than with the placebo, were generally mild and self-limiting. Hyperglycemia was the most common adverse event, particularly in diabetic patients, and was managed through adjustments to diabetes medications. No deaths occurred during the trial, and serious adverse events were rare [8].

The phase III OPTIC study [7] enrolled a total of 83 patients with active TED, who were randomly assigned at a 1:1 ratio to receive either teprotumumab (41 patients) or a placebo (42 patients) through eight instances of infusion. At week 24, the proptosis response rate (defined as a reduction in proptosis of ≥2 mm) was 83% (34/41) in the teprotumumab group, significantly higher than the 10% (4/42) observed in the placebo group, with a number needed to treat (NNT) of 1.36. All secondary endpoints were significantly better in the teprotumumab group compared to those in the placebo group: the overall response rate (a reduction of ≥2 points in the CAS plus a reduction in proptosis of ≥2 mm) was 78% (32/41) vs. 7% (3/42); the proportion of patients with a CAS of 0 or 1 was 59% (24/41) vs. 21% (9/42); the mean change in proptosis was −2.82 mm vs. −0.54 mm; the diplopia response rate (a reduction in diplopia of ≥1 grade) was 68% (19/28) vs. 29% (8/28); and the mean change in the GO-QoL questionnaire scores was 13.79 points vs. 4.43 points (all *p* ≤ 0.001). In addition, among the six patients in the teprotumumab group who underwent orbital imaging, significant reductions in extraocular muscle volume and/or orbital fat volume were observed. Regarding safety, most adverse events were mild or moderate in severity, including hyperglycemia and hearing impairments. Two serious adverse events occurred in the teprotumumab group, one of which (an infusion reaction) led to the discontinuation of treatment [7]. Based on these findings, the FDA granted approval for teprotumumab in January 2020 for the treatment of active TED [6]. According to expert consensus [39], teprotumumab can be used as a first-line treatment for adult patients with TED, especially those with a CAS ≥ 4. Additionally, it may also be considered for patients with a CAS < 3 who have other features such as lid retraction ≥ 2 or mild or early optic neuropathy.

Building upon these pivotal trials, a subsequent pooled analysis [40] of 171 patients from both studies provided critical subgroup insights and long-term durability data. While confirming the overall efficacy (a 77% vs. 15% proptosis response; NNT = 1.6) and a safety profile consistent with the individual trials, this integrated analysis revealed that teprotumumab’s benefits extended uniformly across key subgroups, including older adults (NNT = 1.2 for ≥65 years) and patients grouped by baseline CAS severity and thyrotropin-binding inhibiting immunoglobulin (TBII) levels. Notably, 67% of responders maintained the reduction in proptosis at 51 weeks post-treatment, and 81% achieved a novel composite endpoint (vs. 44% with the placebo; NNT = 2.5) incorporating both inflammatory and structural improvements. The analysis further quantified the quality of life gains (19-point GO-QoL improvement vs. 6-point improvement with the placebo) and identified tobacco users as a subgroup with an attenuated diplopia response (a 29% treatment difference, *p* = 0.086). This pooled analysis demonstrated that teprotumumab treatment significantly ameliorated the clinical course of TED across all subgroups in both trials, with the majority of the patients maintaining durable responses over the extended follow-up period.

The OPTIC-X extension trial [41] conducted later was an open-label clinical extension study designed to evaluate the long-term efficacy and feasibility of repeated treatment with teprotumumab for TED. The study participants included patients with proptosis who did not respond to treatment in the OPTIC trial (defined as less than a 2 mm reduction in proptosis in the studied eye at week 24; *n* = 37 in the placebo group and *n* = 14 in the teprotumumab group). Patients received eight infusions of teprotumumab (the same as in the OPTIC trail). Among the 37 patients who received the placebo in the OPTIC trial, 33 (89.2%) became proptosis responders after receiving teprotumumab treatment in the OPTIC-X study. These patients’ responses were comparable to those in the OPTIC study. Among these responders, maintenance of the reduction in proptosis, a clinical activity score (CAS) of 0 or 1, and a diplopia response were achieved in 90.6%, 95.2%, and 85.7% of the patients, respectively, at week 48 of follow-up. Compared with the patients who received teprotumumab in the OPTIC study, those in the OPTIC-X study had a longer median duration of TED (12.9 months vs. 6.3 months). Among the five patients who did not respond to teprotumumab in the OPTIC trial, two had a response after re-treatment in the OPTIC-X study, with one showing a 1.5 mm reduction in proptosis from the OPTIC baseline, while the other two discontinued treatment early. Among the patients who experienced disease recurrence after receiving teprotumumab in the OPTIC trial, five (62.5%) had a response after re-treatment (with an average proptosis reduction of 1.9 ± 1.2 mm from the OPTIC-X baseline and 3.3 ± 0.7 mm from the OPTIC baseline). Compared with the published double-blind trials and their integrated follow-up studies, no new safety signals were identified. Mild hearing impairments were reported; four events occurred during the first treatment course, with two recurring after re-treatment [41].

### 6.2. Real-World Evidence and Expanding Applications

Following the approval of teprotumumab for the treatment of TED, numerous real-world studies have demonstrated its comparative efficacy and safety profile. Table 2 presents a comprehensive summary of clinical studies investigating teprotumumab in TED.

Immunomodulatory agents such as rituximab and tocilizumab demonstrate efficacy in suppressing immune activation and mitigating inflammatory progression during the active phase of TED [58,59,60]. However, their therapeutic impact on long-term disease outcomes, including proptosis and diplopia, remains limited. Consequently, the conventional management of chronic, non-inflammatory TED with proptosis primarily relies on volume reduction strategies, notably decompression surgery [2,25]. Recent investigations have revealed the consistent overexpression of IGF-1R on the OFs in both active and chronic TED patients [61]. This molecular finding suggests promising therapeutic potential for teprotumumab in the treatment of chronic TED cases. A retrospective study [2] enrolled 31 patients with chronic stable TED (with a mean disease duration of 81 months) who had received ≥ 3 infusions of teprotumumab. The participants received an average of seven teprotumumab injections each. The results demonstrated that teprotumumab treatment significantly improved proptosis, inflammatory signs, diplopia, strabismus, and orbital soft tissue volume in chronic TED patients. The phase IV clinical trial NCT04583735 was an inaugural placebo-controlled trial investigating teprotumumab in chronic/low-disease-activity TED [51], enrolling 62 patients (with 42 receiving teprotumumab and 20 a placebo) with stable TED with a 2- to 10-year duration. At 24 weeks, the teprotumumab group demonstrated superior proptosis reductions compared to those with the placebo (mean change = −2.41 vs. −0.92 mm) while maintaining comparable adverse event profiles. These findings substantiate the therapeutic efficacy and safety of teprotumumab for proptosis management in chronic TED.

Dysthyroid optic neuropathy (DON) represents an uncommon yet severe complication of TED, primarily attributed to mechanical compression of the optic nerves by enlarged orbital tissues in TED patients, which may potentially result in permanent visual impairment [62]. A multicenter observational case series [43] demonstrated that among 10 patients presenting with acute or chronic TED complicated by DON, teprotumumab treatment yielded objective improvements in DON’s manifestations following two infusions in 70% of cases. These improvements encompassed significant visual acuity enhancements, the resolution or amelioration of relative afferent pupillary defects (RAPDs), and restoration or improvement of color vision. Notably, no disease recurrence was observed upon treatment completion. A cross-sectional cohort study [42] also reported that among the 21 TED patients enrolled, 3 cases of DON refractory to methylprednisolone therapy showed improvements following teprotumumab treatment.

Emerging research has increasingly examined the therapeutic efficacy of teprotumumab in atypical or severe cases of TED. A retrospective case series enrolled 26 TED patients with either hypothyroidism or euthyroidism who received eight teprotumumab infusions [55]. Its findings demonstrated significant improvements in both proptosis and inflammatory manifestations, with teprotumumab exhibiting comparable efficacy to the treatment outcomes in GD-associated TED. These results further suggest that IGF-1R pathway dysfunction represents a common pathogenic mechanism in all TED patients, irrespective of thyroid status. A retrospective cohort study conducted in Israel [57] evaluated the therapeutic efficacy of teprotumumab in 32 patients with TED who had demonstrated an inadequate response to prior intravenous glucocorticoid therapy between 2021 and 2024. The study cohort exhibited significant reductions in proptosis, CASs, and diplopia following treatment. Notably, the sample included four patients who had previously undergone orbital decompression surgery (three cases with DON and one case with severe proptosis). Clinical improvements were observed after the administration of one to seven treatment cycles. These findings substantiate the efficacy of teprotumumab in TED patients for whom the standard therapies have previously failed.

Currently, there is very limited research comparing teprotumumab with other TED treatment methods. A meta-analysis compared the therapeutic effects of teprotumumab and IVMP therapy on moderate to severe TED through a matched–adjusted indirect comparison of patients [63]. Compared with the placebo group, the degree of the improvement in baseline exophthalmos in the IVMP therapy treatment group was smaller, while the degree of improvement in exophthalmos in the teprotumumab treatment group was statistically significantly higher than that in the IVMP therapy group (treatment difference: −2.31 mm). Regarding the diplopia response, the IVMP therapy group was not superior to the placebo group, while the teprotumumab group was superior to the IVMP therapy group. The response to diplopia was also better in the teprotumumab group than that in the IVMP therapy group. Another meta-analysis comparing teprotumumab and IVMP therapy (4.5 mg/12 weeks) was conducted to evaluate their effects on QoL in patients with TED [64]. The results demonstrated that teprotumumab significantly improved both the overall GO-QoL scores and its appearance/visual function subscales compared to those with IVMP therapy and the placebo, with mean differences (MD) of 13.26 (95% confidence interval: 7.44, 19.09) and 12.57 (95% confidence interval: 5.94, 19.21), respectively. In contrast, IVMP therapy failed to demonstrate significant improvements in the GO-QoL scores when compared with these scores under the placebo. A retrospective study compared the efficacy and safety of teprotumumab and tocilizumab in treating moderate to severe steroid-resistant TED [49]. The results showed that among the patients with steroid-resistant TED treated with teprotumumab, the proptosis response rate was 81%, the diplopia resolution rate was 45%, and the disease remission rate was 86%, and in 58% of patients, the disease severity was reduced to mild by week 24. Although there was some worsening of proptosis and diplopia from week 24 to week 52, the overall improvement remained better than the baseline. For patients treated with tocilizumab, the proptosis response rate was 50%, the diplopia resolution rate was 17%, and the disease remission rate was 100%, and for 75% of patients, the disease severity was reduced to mild. However, there was a trend of worsening diplopia and disease severity from week 24 to week 52 in the tocilizumab group. In terms of adverse events, 76% of the patients treated with teprotumumab reported adverse events, including audiologic changes (46%) and hyperglycemia (23%), while no adverse events were reported in the tocilizumab group.

Teprotumumab has revolutionized TED management as the first targeted therapy against IGF-1R, demonstrating transformative outcomes across the disease spectrum. Despite these advances, unresolved challenges, including side effects, the re-treatment strategies, and cost-effectiveness, highlight the need for continued research. The following section will critically examine these limitations.

## 7. Current Challenges in Teprotumumab Treatment

### 7.1. Safety Concerns and Adverse Events Profile

Teprotumumab is generally well-tolerated, with the majority of the patients in clinical studies successfully completing the full eight-dose treatment regimen (Table 2). But teprotumumab may cause diverse systemic adverse events (AEs) due to IGF-1R’s widespread expression. For instance, inhibiting IGF-1R reduces the feedback inhibition in growth hormone (GH) secretion, increases GH levels, boosts glucose production and insulin resistance, and thus causes hyperglycemia. Also, it can weaken the survival signals of the inner-ear hair cells and support cells, reduce their quantity, and trigger adverse otic reactions [65].

According to an integrated analysis of the phase II and III OPTIC trials [8,40,41,50], during the treatment period, 80% (67/84) of patients receiving teprotumumab experienced adverse reactions, among whom three (4%) had severe adverse events related or possibly related to teprotumumab, including diarrhea, infusion reactions, and Hashimoto’s encephalopathy (with confusion). The remaining adverse events were mild to moderate (grade 1 or 2). In the placebo group, 70% (60/87) of patients experienced adverse reactions, including one severe adverse event. Compared with the placebo, teprotumumab use was associated with an increased risk of muscle spasms (an 18% higher risk, 95% CI 7.3–28.7), hearing impairments (a 10% higher risk), and hyperglycemia (an 8% higher risk, 95% CI 1.7–15.0). Among the patients with hyperglycemia, five had a history of diabetes. All hyperglycemic events were resolved during the treatment period or after the final dose. Most of the hearing loss events were classified as non-severe, and all patients continued the study without event exacerbation. During the 48-week follow-up period, 29 (39%) of 74 patients in the teprotumumab group reported adverse events, compared with 9 (12%) of 74 in the placebo group. Onychoclasis was the most common adverse event in the teprotumumab group (9% vs. 0% in the placebo group). During the follow-up period, 2 (3%) of 74 patients in the teprotumumab group reported severe adverse events (intercostal neuralgia and optic neuropathy, which were considered unrelated to the study drug), while 1 (2%) of 46 patients in the placebo group reported a severe adverse event. Based on the aforementioned data, the FDA has classified the principal AEs associated with teprotumumab as follows: infusion-related reactions (manifesting as transient hypertension, pyrexia, tachycardia, dyspnea, cephalalgia, and myalgia, with an incidence rate of approximately 4%); hyperglycemia or elevated blood glucose levels (documented in approximately 10% of study participants); auditory disturbances, including hearing impairment (affecting nearly 8% of recipients); and exacerbation of pre-existing inflammatory bowel disease (IBD) [40,52,66].

While randomized controlled trials have provided controlled efficacy and safety data, real-world evidence has significantly expanded our understanding of teprotumumab’s adverse effect profile in diverse clinical settings. Table 3 summarizes the real-world adverse events associated with teprotumumab treatment for TED.

A recently published retrospective observational cohort study led by Shah et al. involving 131 TED patients treated with teprotumumab provided a more comprehensive evaluation of the incidence, duration, reversibility, and severity of the adverse events associated with teprotumumab therapy [52]. This study found that 81.7% of patients experienced at least one treatment-related AE, with a median of four AEs per patient. Most AEs were mild (74.0%), but 28.2% were moderate and 8.4% severe. The most common AEs were musculoskeletal (58.0%), followed by gastrointestinal (38.2%), skin (38.2%), ear and labyrinth (30.5%), nervous system (20.6%), metabolic (15.3%), and reproductive system (12.2%) issues. Notably, 46% of patients had at least one persistent AE at the last follow-up, and 12.2% of the patients discontinued treatment due to AEs, primarily related to hearing loss, IBD, and hyperglycemia. In this cohort, approximately one-third of patients exhibited auditory symptoms or hearing impairments, which may have been associated with the relatively advanced mean age of the study population. In addition, the two patients who developed IBD during treatment in this cohort were both new cases rather than exacerbations of pre-existing IBD [52].

Global research has identified ethnicity as a potential risk factor for teprotumumab-induced hyperglycemia. A longitudinal observational study investigating the glycemic changes following teprotumumab treatment for TED revealed that Hispanic and Asian ethnicities constitute significant risk factors for hyperglycemia [67]. In the first Japanese randomized controlled trial (RCT) cohort undergoing teprotumumab treatment, the incidence of hyperglycemia (22%) exceeded that reported in the phase II and III trials (pooled analysis: 10%) [40,54]. This discrepancy may be attributed to the underrepresentation of Asian participants (3/103) in the latter studies [7,8,40,66].

Furthermore, there have been reports of rare but severe cases of teprotumumab-induced encephalopathy [57,68,69], characterized by rapidly progressive cognitive impairments. These patients demonstrated symptomatic improvements following therapeutic plasma exchange.

### 7.2. Economic Burden and Cost-Effectiveness Considerations

One of the primary limiting factors hindering the widespread adoption of teprotumumab is its substantial cost burden. The cost of a single course of teprotumumab treatment is approximately USD 360,000 [70]. Shah et al. [71] conducted a cost-effectiveness analysis comparing teprotumumab with alternative therapeutic approaches to TED, employing the validated GO-QoL questionnaire to measure the QoL improvements. The investigators extracted the treatment costs from the Turquoise Health database using Medicare billing codes, with all of the costs standardized to a 70 kg patient. The findings demonstrated that teprotumumab incurred the highest mean treatment-related costs (USD 386,424) and yielded the greatest improvement in GO-QoL scores (ΔGO-QoL = 19.4). However, it also exhibited the least favorable cost/ΔGO-QoL ratio (USD 19,888), representing approximately 20-fold and 50-fold increases compared to those for rituximab and IVMP, respectively.

Researchers are endeavoring to investigate the efficacy of abbreviated treatment regimens to mitigate the financial burden associated with teprotumumab. A retrospective cohort study conducted in Israel [57] evaluated the therapeutic outcomes of teprotumumab in 32 TED patients who had previously demonstrated an inadequate response to intravenous glucocorticoid therapy between 2021 and 2024. The findings revealed that 40.6% of cases exhibited rapid improvements in their diplopia and proptosis symptoms following just 2–3 treatment administrations, suggesting that a shorter treatment regimen may be efficacious for certain TED patients. In another study involving 74 patients, including 62 with active disease and 12 with minimal or no clinical activity, treatment with teprotumumab was interrupted after an average of 4.2 infusions due to the COVID-19 pandemic [50]. Nevertheless, both groups demonstrated significant improvements in proptosis (a mean reduction of 2.9 mm in active patients versus 2.8 mm in minimally/non-active patients, *p* < 0.01), with the therapeutic effects sustained during the mean follow-up period of 13.2 weeks after treatment cessation. Patients with active disease exhibited a mean reduction of 3.4 points in their CASs (*p* < 0.01) and improved ocular motility (*p* < 0.01), with these benefits persisting throughout the follow-up period. These findings suggest that even partial courses of teprotumumab treatment can yield meaningful clinical benefits for TED patients.

### 7.3. Treatment Durability and Insufficient Long-Term Data

Currently, there remains a paucity of long-term clinical experience with teprotumumab and limited post-treatment follow-up data. A retrospective study indicated the possibility of proptosis recurrence after the discontinuation of treatment [72]. The results showed that, after an average follow-up of 10.56 months, among 78 patients who had multiple follow-up visits, 102 eyes of 59 patients (65.38%) experienced proptosis recurrence after treatment discontinuation. The ongoing phase IIIb/IV clinical trial NCT05002998 [73] is investigating the therapeutic efficacy and re-treatment requirements across three distinct treatment regimens (4 infusions, 8 infusions, and 16 infusions) in patients with TED. Initially registered on 19 July 2021, this study is projected to conclude in 2026. However, currently, as an FDA-approved drug, the long-term data on teprotumumab remains insufficient, which increases its potential risks in clinical application.

## 8. Other Emerging IGF-1R Inhibitors for TED

Beyond teprotumumab, several other IGF-1R-targeting therapeutics are currently under development. VRDN-001, a monoclonal antibody against IGF-1R, is undergoing phase I/II clinical trials (NCT05176639). Administered via intravenous infusion at doses of 3 mg/kg or 10 mg/kg every three weeks for two cycles, preliminary results demonstrate a mean reduction in proptosis of 1.5 mm (a 33% response rate, a 2.0-point CAS improvement) in a 3 mg/kg cohort and 1.8 mm (a 50% response rate, a 1.8-point CAS improvement) in a 10 mg/kg cohort, with no serious adverse events reported [74].

VRDN-002 and VRDN-003 are modified IGF-1R antibodies engineered for an extended half-life. VRDN-002 incorporates Fc region modifications, achieving an approximately fourfold prolongation of its half-life. In a phase I trial involving healthy volunteers, a single 300 mg/2 mL intravenous or subcutaneous dose induced elevated serum IGF-1 levels. VRDN-003 demonstrated a twofold longer half-life than that of VRDN-001 in preclinical primate studies following both intravenous and subcutaneous administration, suggesting potential for sustained therapeutic concentrations with low-volume subcutaneous dosing [75,76].

Linsitinib, a small-molecule IGF-1R inhibitor, is being evaluated in a phase IIb trial (NCT05276063) with twice-daily oral administration over six months. Murine models indicate its efficacy in preventing autoimmune hyperthyroidism and reducing orbital immune cell infiltration [77]. And Lonigutamab, a high-affinity anti-IGF-1R monoclonal antibody, is currently in phase I/II trials, with subcutaneous dosing regimens including administration every 3 weeks (two doses of 40 mg) and weekly administration (a 50 mg loading dose followed by 25 mg for 11 weeks) [78].

## 9. Limitations

This review has several methodological limitations that warrant consideration. A critical challenge in synthesizing the evidence across studies was the substantial heterogeneity in the research methodologies. The included studies varied considerably in their design (from randomized controlled trials to retrospective case series), sample size, outcome measures, and follow-up durations. This heterogeneity complicates direct comparisons and limits the strength of the conclusions that can be drawn.

The pivotal clinical trials employed stringent inclusion criteria that may not reflect the diversity of the patients encountered in clinical practice. For instance, patients with poorly controlled diabetes, significant hearing impairments, and IBD were excluded from the phase II and III trials, yet real-world studies have subsequently included such patients, with varying results. This discrepancy between trial populations and real-world patients creates challenges in generalizing the efficacy and safety findings.

Additionally, the outcome measures varied substantially across studies. While proptosis reductions and clinical activity scores were commonly reported, the assessment of diplopia, quality of life, and adverse events lacked standardization. Some studies employed validated instruments like the Graves’ Orbitopathy-Specific Quality of Life questionnaire, while others used non-validated or subjective measures, limiting cross-study comparisons.

The absence of data regarding teprotumumab use in pediatric populations and pregnant or lactating women represents another significant knowledge gap in our analysis. Furthermore, publication bias may exist, as negative results are less likely to be published, potentially overestimating the treatment efficacy.

Despite these limitations, this review synthesizes the best available evidence to provide a comprehensive assessment of teprotumumab’s role in TED management while acknowledging areas requiring further investigation.

## 10. Conclusions and Future Directions

Teprotumumab represents a paradigm shift in TED management as the first FDA-approved targeted therapy addressing its underlying pathophysiology through IGF-1R inhibition. Our critical analysis yields three key conclusions: First, teprotumumab demonstrates remarkable clinical efficacy across diverse TED populations. Pivotal trials and real-world evidence consistently show significant improvements in proptosis, CASs, and diplopia, with the benefits extending to both active and chronic disease states. Notably, teprotumumab shows promise in complex cases, including steroid-resistant disease and dysthyroid optic neuropathy. Second, despite its transformative efficacy, teprotumumab presents significant implementation challenges. The treatment is associated with specific adverse effects—particularly hyperglycemia and hearing impairments—with emerging evidence suggesting ethnic variations in susceptibility. Its substantial cost creates accessibility barriers and raises cost-effectiveness concerns compared to conventional therapies. Third, critical knowledge gaps persist regarding its optimal clinical application. Limited long-term follow-up data, disease recurrence in 65% of patients, and insufficient evidence for re-treatment protocols highlight the need for extended monitoring studies. The absence of research comparing its effectiveness against that of established therapies further complicates clinical decision-making.

Ongoing clinical trials investigating modified treatment protocols, along with recent explorations of abbreviated treatment courses, may address some of these challenges. Future research should prioritize optimizing the patient selection criteria, developing strategies to mitigate adverse effects, establishing re-treatment protocols, and conducting comprehensive long-term safety and efficacy assessments to define teprotumumab’s position within the TED treatment paradigm better.

## Figures and Tables

**Table 1:** A summary of the traditional clinical considerations in the management of thyroid eye disease (TED) (based on the European Group on Graves’ Orbitopathy (EUGOGO)’s clinical recommendations; Bartalena et al., 2021 [32]).

Treatment Phase	Disease Stage	Therapeutic Approach	Intervention	Clinical Indications	Key Considerations
**Initial Management**	All Stages	Foundational Measures	Confirm Graves’ disease (TRAb testing) Achieve euthyroidism (prioritize hyper-/hypothyroid control)	All newly diagnosed TED cases	Thyroid stability is imperative Requires periodic reassessment
		Ocular Support	Artificial tears, nocturnal protection for corneal exposure prophylaxis	Universal TED presentation	Heightened vigilance for keratopathy
		Behavioral Modification	Smoking cessation with structured support Minimize passive smoke exposure	All TED patients	Tobacco use exacerbates disease progression
		Specialist Consultation	Referral to TED multidisciplinary center	Moderate to severe or progressive disease	Facilitates timely, tailored management
**Mild TED**	Active Phase	Pharmacotherapy	Selenium supplementation (100 μg BID × 6 months)	Mild active TED, particularly in endemic selenium-deficient areas	Potential prostate cancer association Avoid supratherapeutic dosing
		Surveillance	Serial clinical activity scores (CASs)	Stable, minimally symptomatic presentations	Escalate therapy if progression occurs
	Inactive Phase	Reconstructive Intervention	Eyelid repositioning surgery	Persistent retraction or cosmetic impairment	Requires ≥ 6 months of thyroid stability
**Moderate-to-Severe TED**	Active Phase	Intravenous Corticosteroids	Methylprednisolone (cumulative 4.5 g: 0.5 g/week × 6, then 0.25 g/week × 6)	Active inflammatory moderate to severe TED	Terminate if non-responsive by 6 weeks Hepatic/glucose monitoring essential
		Biologic Agents	Rituximab Tocilizumab	Rituximab: steroid-refractory cases with marked inflammation/diplopia progression Tocilizumab: glucocorticoid failure	Limited longitudinal data on efficacy
		Radiotherapy	Fractionated orbital RT (20 Gy/10 fractions) ± glucocorticoids	Progressive ocular motility impairment or steroid contraindications	Caution in diabetics May transiently worsen periorbital edema
	Inactive Phase	Surgical Correction	Orbital decompression (proptosis reduction) Strabismus surgery (diplopia management) Eyelid reconstruction	Quiescent disease (≥6 months) with residual functional/aesthetic deficits	Requires individualized sequencing Standard approach: decompression → strabismus → eyelid
**Sight-Threatening TED**	Active Phase	Urgent Medical Therapy	High-dose IV methylprednisolone (0.75–1 g weekly × 2)	Dysthyroid optic neuropathy or acute visual decline	Surgical decompression if inadequate response within 2 weeks
		Emergency Surgery	Extensive medial/inferior wall decompression	Vision deterioration despite medical therapy	Requires subspecialty orbital surgical expertise
		Corneal Salvage	Intensive lubrication, antibiotic prophylaxis Temporary tarsorrhaphy	Impending corneal perforation or ulceration	Surgical delay risks irreversible visual loss

**Table 2:** Summary of clinical studies on teprotumumab for thyroid eye disease after FDA approval.

Study Design	Sample Size	Patient Characteristics	Treatment Dosage	Primary Outcomes	Secondary Outcomes	Notes	Study
Cross-sectional cohort	21	Heterogeneous TED including three DON cases	8 doses	71.4% achieved ≥2 mm proptosis reduction	CAS −2.2, motility +16.9°	DON cases improved	Diniz et al., 2021 [42]
Multicenter case series	10	DON patients in whom conventional therapy failed	8 infusions	VA improvement = 0.87 logMAR	Proptosis −4.7 mm, CAS −5.25	Rapid DON improvement	Sears et al., 2021 [43]
Prospective longitudinal	23	Patients with TED accompanied by facial/eyelid changes	8 infusions	Reduction in facial soft tissue volume	Improvement in eyelid position	Orbital soft tissue expansion was also reduced	Ugradar et al., 2021 [44]
Retrospective EAP study	13	Active moderate–severe TED	8 infusions (77%)	Proptosis −4.6 mm	CAS −4.0, light sensitivity −9.1	Expanded access program	Wang et al., 2021 [45]
EAP study	22	Active moderate–severe TED	8 infusions (86%)	QOL improvement	Not specified	All patients reported AEs; mainly muscle spasms (*n* = 11), fatigue (*n* = 10)	Douglas et al., 2021 [46]
Prospective longitudinal	43	Active TED with facial changes	8 infusions	Facial volume reduction (mean decrease = 8.4 mL)	Proptosis improvement	Pan-facial assessment	Ugradar & Douglas, 2022 [47]
Retrospective review	31	Chronic TED (>2 years)	Mean: 7 infusions	Proptosis −3.5 mm	67% diplopia improvement	Teprotumumab treatment had therapeutic efficacy in patients with chronic TED	Ugradar et al., 2022 [2]
Retrospective review	17	TED with IOP concerns	Mean: 12 weeks	Mean IOP was decreased at last record of follow-up by 4.9 mm Hg	Not specified	Teprotumumab treatment reduced IOP	Adetunji et al., 2022 [48]
Retrospective study	37	Steroid-naive and steroid-resistant TED	Up to 8 infusions	81% proptosis response in steroid-resistant group	45% diplopia resolution, 86% disease inactivation	Comparison with tocilizumab	Kotwal et al., 2023 [49]
Observational cross-sectional	74	Active (*n* = 62) and minimal-activity (*n* = 12) TED; treatment interrupted	Average of 4.2 infusions	Proptosis: −2.9 mm (active), −2.8 mm (minimal)	CAS −3.4	COVID-19 interrupted this study	Ho et al., 2023 [50]
RCT	62	Chronic/low-activity TED (2–10 years)	8 infusions	Proptosis: −2.41 mm vs. −0.92 mm(placebo)	Not specified	First chronic placebo-controlled trial with TED	Douglas et al., 2023 [51]
Multicenter retrospective	131	All stages/activity levels of TED	≥4 infusions	76% achieved proptosis −3.0 mm	3.2-point average CAS reduction, GDS improved by at least 1 point for 50%	Comprehensive AE study	Shah et al., 2024 [52]
Multicenter retrospective	66	Recalcitrant TED for which conventional therapy failed	≥4 infusions	85.9% proptosis response	CAS 93.8%, diplopia 69.1%	Poor response post-decompression	Men et al., 2024 [53]
RCT	54	Active moderate–severe TED in Japanese cohort	8 infusions	89% vs. 11% (placebo) proptosis responses	78% vs. 4% (placebo) overall responses	First Japanese RCT	Hironmatsu et al., 2025 [54]
Multicenter case series	26	Hypothyroid/euthyroid TED	8 infusions	Proptosis −2.7 mm	CAS and diplopia improvements	Non-hyperthyroid TED	Ugradar et al., 2025 [55]
Multicenter retrospective	119	Complete treatment with 1-year follow-up	8 infusions	24% re-treatment rate	Not specified	Age was the only significant driver of re-treatment	Ugradar et al., 2025 [56].
Retrospective cohort study	32	Failed intravenous glucocorticoid treatment; four with prior decompression surgery	8 infusions	Proptosis: R −2.4 mm, L −2.0 mm	Improvement in diplopia	One case of teprotumumab-induced encephalopathy was reported and successfully treated using plasma exchange; decompression surgery history did not affect efficacy	Lustig-Barzelay et al., 2025 [57]

Studies are listed chronologically and include the study design, patient characteristics, treatment protocols, and efficacy outcomes. AE = adverse event; CAS = clinical activity score; DON = dysthyroid optic neuropathy; EAP = expanded access program; GDS = Gorman diplopia score; IOP = intraocular pressure; QOL = quality of life; RCT = randomized controlled trial; TED = thyroid eye disease; VA = visual acuity.

**Table 3:** Real-world adverse event profile for teprotumumab in thyroid eye disease treatment.

Author (Year)	Patients Treated with Teprotumumab	Total AE Rate	Common AEs (>10%)	Serious AEs	Special Notes
Ho et al., 2023 [50]	74	Initial: 66% Final: 29%	- Muscle spasms (27%) - Alopecia (18%) - Hyperglycemia (14%) - Hearing changes (11%) - Fatigue (9%) - GI discomfort (8%)	Three new diabetes cases; one severe hyperglycemia case (>700 mg/dL)	- AEs decreased over time - No new DON cases during interruption - One patient discontinued treatment due to hyperglycemia—three patients required oral diabetes medication
Diniz et al., 2021 [42]	21	85.7%	- Fatigue (43%) - Muscle spasms (33%) - Dysgeusia (26%) - Nausea (19%) - Weight loss (14%) - Hearing issues (14%) - Hyperglycemia (14%)	Three cases requiring diabetes medication	- Most AEs were of a grade 1–2 severity - One discontinuation due to multiple AEs - Two/three hyperglycemia cases in non-diabetic patients - Age may be risk factor for hearing issues
Lustig-Barzelay et al., 2025 [57]	32	Not specified	- Myalgia (*n* = 12%) - Hyperglycemia (*n* = 9%) - Diarrhea (*n* = 9%) - Hearing issues (*n* = 12%)	One case of encephalopathy	- Encephalopathy successfully treated with plasmapheresis - Real-world Israeli cohort experience
Douglas et al., 2021 [46]	22	100%	- Muscle spasms (50%) - Fatigue (45%) - Hypoacusis (23%)–headache (23%) - Nausea (23%) - Extremity pain (18%)–alopecia (18%) - Hypertension (18%)	One case of appendicitis (deemed unrelated)	- Multiple other AEs reported in smaller numbers (*n* = 3): dry skin, diarrhea, tinnitus, myalgia, increased lacrimation, hypogeusia
Kotwal et al., 2023 [49]	37	76%	- Hearing changes (46%) - Hyperglycemia (23%)	Not specified	- Compared with the tocilizumab group which reported no AEs
Shah et al., 2024 [52]	131	82%	- Musculoskeletal (58.0%) - GI (38%) - Skin (38%) - Ear/hearing (31%) - Nervous system (21%) - Metabolic (15%)–reproductive (12%)	8.4% (11/131) severe AEs	- Mean AE onset: 7.9 weeks - Mean duration: 17.6 weeks - 46% had persistent AEs at last follow- up - 12.2% discontinued therapy (hearing loss *n* = 4, IBD *n* = 2, hyperglycemia *n* = 1, muscle spasms *n* = 1, multiple AEs *n* = 8)
Hironmatsu et al., 2025 [54]	27	Not specified	- Hyperglycemia (22%) - Hearing impairment (15%)	Not specified	- The Japanese population - Placebo-controlled comparison available

Abbreviations: AE: Adverse Event, GI: Gastrointestinal, DON: Diabetic Ocular Neuropathy, IBD: Inflammatory Bowel Disease.

## Data Availability

Not applicable.
